# Engineering the xylose metabolism in *Schizochytrium* sp. to improve the utilization of lignocellulose

**DOI:** 10.1186/s13068-022-02215-w

**Published:** 2022-10-26

**Authors:** Ling-Ru Wang, Zi-Xu Zhang, Fang-Tong Nong, Jin Li, Peng-Wei Huang, Wang Ma, Quan-Yu Zhao, Xiao-Man Sun

**Affiliations:** 1 School of Food Science and Pharmaceutical Engineering, Nanjing Normal University, 2 Xuelin Road, Qixia District, Nanjing, Jiangsu China; 2 School of Pharmaceutical Science, Nanjing Tech University, No. 30 Puzhu South Road, Pukou District, Nanjing, Jiangsu China

**Keywords:** *Schizochytrium* sp., Xylose metabolism, Lignocellulose, Metabolic engineering, Lipid production

## Abstract

**Background:**

*Schizochytrium* sp. is a heterotrophic, oil-producing microorganism that can efficiently produce lipids. However, the industrial production of bulk chemicals using *Schizochytrium* sp. is still not economically viable due to high-cost culture medium. Replacing glucose with cheap and renewable lignocellulose is a highly promising approach to reduce production costs, but *Schizochytrium* sp. cannot efficiently metabolize xylose, a major pentose in lignocellulosic biomass.

**Results:**

In order to improve the utilization of lignocellulose by *Schizochytrium* sp., we cloned and functionally characterized the genes encoding enzymes involved in the xylose metabolism. The results showed that the endogenous xylose reductase and xylulose kinase genes possess corresponding functional activities. Additionally, attempts were made to construct a strain of *Schizochytrium* sp. that can effectively use xylose by using genetic engineering techniques to introduce exogenous xylitol dehydrogenase/xylose isomerase; however, the introduction of heterologous xylitol dehydrogenase did not produce a xylose-utilizing engineered strain, whereas the introduction of xylose isomerase did. The results showed that the engineered strain 308-XI with an exogenous xylose isomerase could consume 8.2 g/L xylose over 60 h of cultivation. Xylose consumption was further elevated to 11.1 g/L when heterologous xylose isomerase and xylulose kinase were overexpressed simultaneously. Furthermore, cultivation of 308-XI-XK(S) using lignocellulosic hydrolysates, which contained glucose and xylose, yielded a 22.4 g/L of dry cell weight and 5.3 g/L of total lipid titer, respectively, representing 42.7 and 30.4% increases compared to the wild type.

**Conclusion:**

This study shows that engineering of *Schizochytrium* sp. to efficiently utilize xylose is conducive to improve its utilization of lignocellulose, which can reduce the costs of industrial lipid production.

**Supplementary Information:**

The online version contains supplementary material available at 10.1186/s13068-022-02215-w.

## Background

*Schizochytrium* sp. is a heterotrophic marine fungus with an excellent lipid accumulation ability [1], reaching over 50% of the dry cell weight (DCW) under specific cultivation conditions [2]. The lipid composition of *Schizochytrium* sp. is simple, mainly including myristic acid (C14:0), palmitic acid (C16:0), docosapentaenoic acid (DPA) and docosahexaenoic acid (DHA) [3]. Myristic acid and palmitic acid can be used as biodiesel, and DHA is an essential fatty acid for human health. Moreover, in addition to fatty acids, *Schizochytrium* sp. is also considered as a good choice for squalene and carotenoids production [4, 5]. Therefore, reducing the fermentation cost of *Schizochytrium* sp. is great significance for their further development and application.

In recent years, industrial production based on *Schizochytrium* sp. has been expanding, especially for high-value products (e. g. DHA). For example, William et al. [6] screened a series of *Schizochytrium* sp. and *Thraustochytrids* strains in ocean, which were optimized for subsequent production by Omega Tech. Similarly, *Schizochytrium* sp. has been widely used in the production of DHA algae oil, with a production capacity of about 100,000 tonnes in 2004 [7]. However, in the industrial fermentation processes of *Schizochytrium* sp., the cost of raw materials still makes up a sizeable fraction of total costs, especially the organic carbon sources. According to reports, the cost of glucose makes up more than 73% of the overall cost when it is used as the primary carbon source [8]. As a result, numerous studies have tried to lower the cost of producing *Schizochytrium* sp. by substituting glucose with less expensive carbon sources. For example, when waste glycerol was used as the organic carbon source for the fermentation of *Schizochytrium* sp., 66.7 g/L of DCW and 17.3 g/L of DHA were obtained [9]. By optimizing the culture condition, *Schizochytrium limacinum* SR21 was able to produce 146 g/L of biomass and 82.3 g/L of lipids using acetic acid as the primary carbon source [10]. Yin et al. [11] used sugarcane molasses as a low-cost carbon source to replace glucose in fermentation medium for *Schizochytrium* sp., and the final titer of DHA reached 15.2 g/L after optimization of the fermentation conditions. These studies demonstrate that waste biomass resources can be used as an alternative carbon source by *Schizochytrium* sp. to produce lipids.

The annual global primary production of biomass is about 220 billion tonnes on dry weight basis per year [12]. It primarily comes from waste products from food industry, agriculture, municipal solid trash, and other sources. Utilizing these wastes to create some value-added products is advantageous for the environment as well as economically because dumping them in the ground or in landfills might result in major environmental issues [13]. With the synthesis of bioethanol from lignocellulose as a feedstock already becoming a well-established commercial technique, the production of high-value chemicals from lignocellulose has shown to be economically feasible [14, 15]. Lignocellulose is a promising renewable resource since it is plentiful and easily accessible [16–19]. However, there are two main limitations in the efficient utilization of lignocellulose by microorganisms. Firstly, growth inhibitors are generated during the pretreatment of lignocellulose, which can severely affect the fermentation performance. Secondly, the majority of microorganisms cannot efficiently utilize xylose for growth, despite the fact that it is the second most prevalent monosaccharide in lignocellulosic hydrolysates after glucose. To overcome the first obstacle, adaptive evolution strategies are frequently applied to increase the tolerance of microorganisms to lignocellulosic degradation products. Recently, *Schizochytrium* sp. mutants with great tolerance to hydrolysis by-products have been developed using adaptive laboratory evolution [20]. Moreover, detoxification techniques are often used to lower the inhibitor concentration in the hydrolysate [21]. To address the second obstacle, researchers have concentrated on constructing or enhancing xylose metabolic pathways to increase xylose utilization. To make xylose usable, it is frequently introduced into *Corynebacterium glutamicum*, *Saccharomyces cerevisiae*, *Yarrowia lipolytica*, and *Propionibacterium freudenreichii* via the heterologous xylose reductase–xylitol dehydrogenase pathway or xylose isomerase pathway [22–25]. However, there are still few reports on xylose utilization by *Schizochytrium* sp., necessitating further exploration.

The biomass of *Thraustochytrium* MAN37 was 3.1 g/L at 72 h and that of *Aurantiochytrium* sp. was 1.7 g/L at 120 h when cultured in a medium with xylose as the main carbon source, while thraustochytrid T18 could not grow in a medium containing xylose as the only carbon source [26–28]. These results indicate that different *thraustochytrids* species have differing capacities for utilizing xylose. *Schizochytrium* sp. HX-308 has a highly effective capacity for lipid synthesis. Its DCW and total lipid can reach 165.0 and 113.6 g/L, respectively, under specific circumstances [29]. Our previous research had shown that HX-308 has relatively high tolerance to furfural derivatives and organic acids found in lignocellulosic hydrolysates [30]. However, HX-308 is unable to use xylose to safeguard cell growth, which limits the utilization of lignocellulose. Here, we examined the functional activity of enzymes related to the xylose metabolism pathway of HX-308 in vitro. Furthermore, heterologous genes were introduced into HX-308 to obtain the engineered strain that can utilize xylose. Finally, the lignocellulose utilization ability of the resulting engineered strains was characterized in detail.

## Results

### In vitro activity of enzymes encoded by native xylose metabolic pathway genes of HX-308

**Fig. 1:**
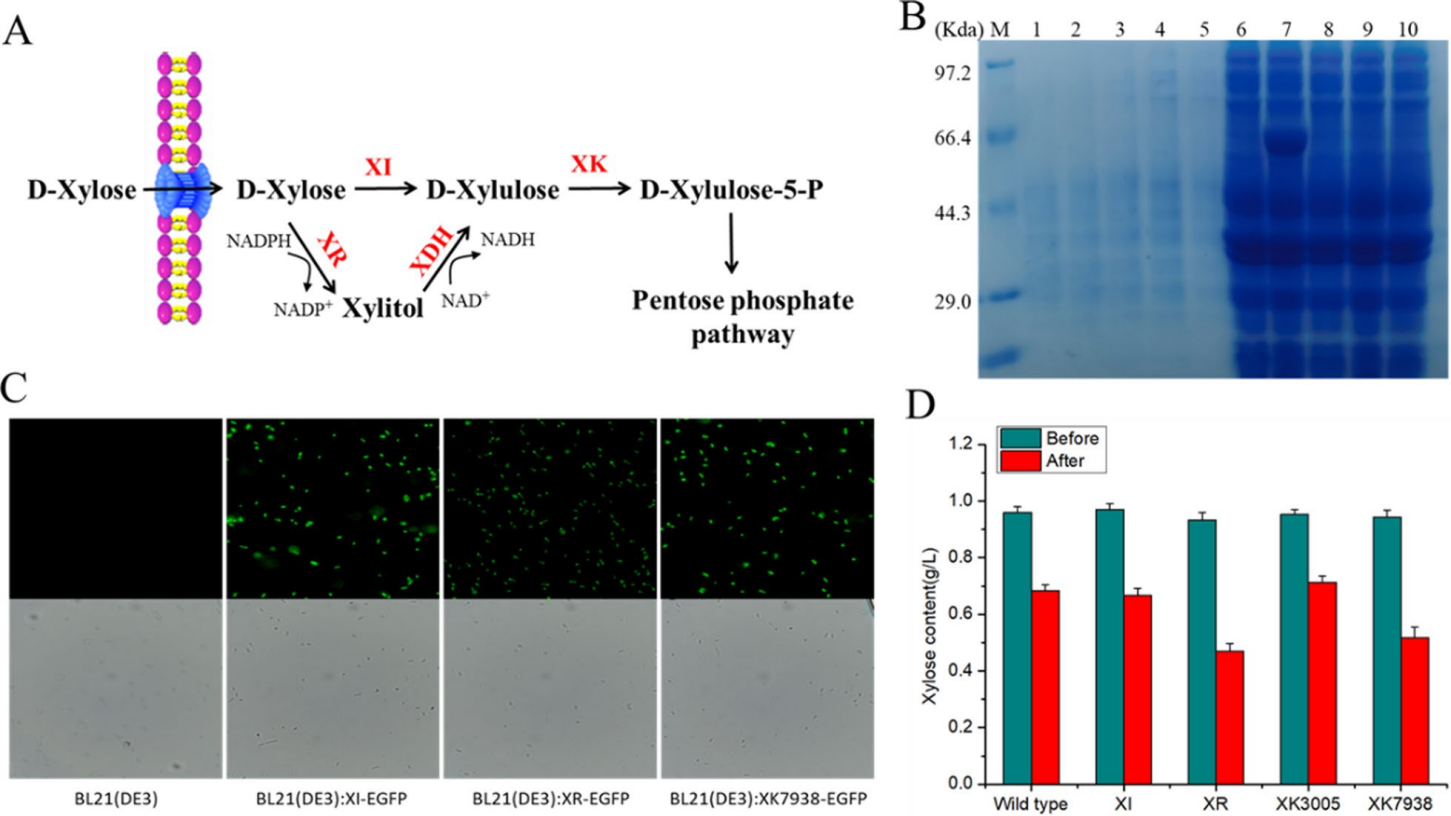
**A** Two xylose metabolism pathways in microorganisms. **B** Protein expression of four key genes involved in xylose metabolism of HX308. Lines 1–5: extracellular protein: Control, XK3005, XK7938, XI, XR; Lines 6–10: intracellular protein: Control, XK3005, XK7938, XI, XR. **C** Fluorescence diagram of strains containing three fusion proteins. **D** Xylose content of the crude enzyme solution system before and after incubation. *XI* xylose isomerase, *XR* xylose reductase, *XDH* xylitol dehydrogenase, *XK* xylulose kinase. Data represent the mean ± standard deviation (*n* = 3)

Fungi mainly utilize xylose via the redox pathway, while bacteria utilize the isomerase pathway [31, 32] (Fig. 1A). The xylulose produced by both pathways is converted into xylulose-5-phosphate by xylulokinase (XK) and channeled into the pentose phosphate pathway. In this study, multiple genes associated with the xylose metabolism pathway were identified from HX-308 by BLASTp alignment (from *Hondaea fermentalgiana*), including xylose isomerase (XI), xylose reductase (XR), as well as two XKs (XK3005 and XK7938) (Additional file 1). To further verify whether these four genes encode corresponding functional enzymes, the four encoded enzymes were cloned in *Escherichia coli*. However, only the XK3005 protein was successfully expressed as a soluble protein (Fig. 1B). Regrettably, the XI, XR and XK7938 proteins had no soluble expression even when the temperature was reduced from 28 to 16 °C [33]. It has been reported that fusing the enhanced green fluorescent protein (eGFP) to the target proteins could facilitate their expression [34–36]. Therefore, the eGFP was, respectively, fused to the C-terminus of the XI, XR and XK7938 proteins, and results showed that the fluorescence was successfully detected compared to the control strain (Fig. 1C). In this study, since the *E. coli* does not contain the XR–XDH pathway which avoids XI activity inhibition by xylitol, and does not need to add additional NADPH required in XR catalysis, then the related engineered *E. coli* extraction was used to test the enzyme function. As shown in Fig. 1D, when the resulting crude enzyme solution was incubated with xylose, the xylose content of the XI and XK3005 group did not decrease compared to the control, indicating that these two enzymes were inactive. However, the xylose consumption of the crude enzyme solution containing the XR and XK7938 proteins was, respectively, increased by 67.5 and 54.2%, compared to the control (Fig. 1D), confirming that these two proteins possess the predicted activity.

### Engineering a complete xylose redox pathway in HX-308

**Fig. 2:**
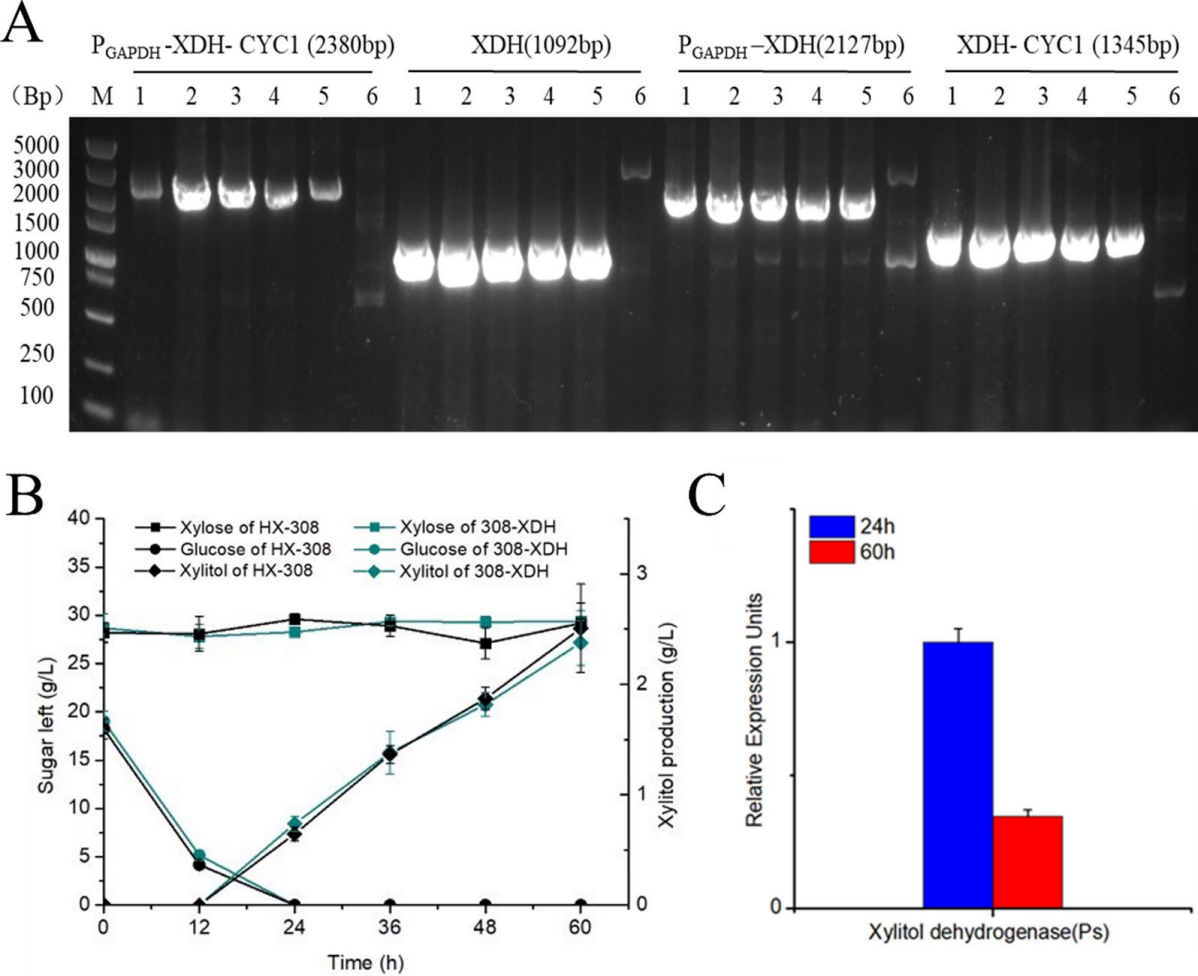
**A** PCR analysis of the 308-XDH. 1–5: XDH transformant, 6: wild type. **B** Time-course determination of residual glucose (g/L), residual xylose (g/L) and xylitol concentration (g/L) of HX-308 and 308-XDH cultivated in medium with 20 g/L glucose and 30 g/L xylose. **C** Quantification of the transcription levels of PsXDH in 24 h and 60 h cultivated in medium with 30 g/L glucose and 20 g/L xylose. 24 h represents the period of glucose consumption and 60 h represents the period of xylose consumption. Data represent the mean ± standard deviation (*n* = 3)

The construction of a complete xylose metabolic pathway is essential for microbial utilization of xylose. Accordingly, we introduced the xylitol dehydrogenase (XDH) gene of *Pichia pastoris* (PsXDH), which is well worked in *S. cerevisiae* [37], to complement the xylose redox pathway in HX-308. We chose to construct the PsXDH expression cassette using the promoter of the housekeeping gene glyceraldehyde-3-phosphate dehydrogenase in order to effectively express PsXDH [38]. The expression cassette of the PsXDH gene, controlled by a strong promoter, was randomly integrated into the HX-308 genome by an *Agrobacterium*-mediated transformation method, resulting in the chromosomally engineered strain 308-XDH (Fig. 2A). However, there was no significant increase of biomass when 308-XDH was cultured in a medium with xylose as the sole carbon source. Moreover, when 308-XDH was cultured in a mixed sugar medium containing 20 g/L glucose and 30 g/L xylose, the xylose concentration in the medium still did not change significantly (Fig. 2B). Additionally, there was no significant difference in the amount of xylitol compared to the wild type. As a result, we speculate that 308-XDH's failure to utilize xylose may be due to the low XR activity of *Schizochytrium* sp. Notably, the transcript level of PsXDH was 66% lower at 60 h (after glucose depletion) than that of 24 h (before glucose depletion) (Fig. 2C). It is possible that PsXDH's ineffective expression is also to blame for 308-XDH's ineffective xylose use.

### Overexpression of a heterologous XI gene to obtain a *Schizochytrium* sp. strain capable of xylose utilization

**Fig. 3:**
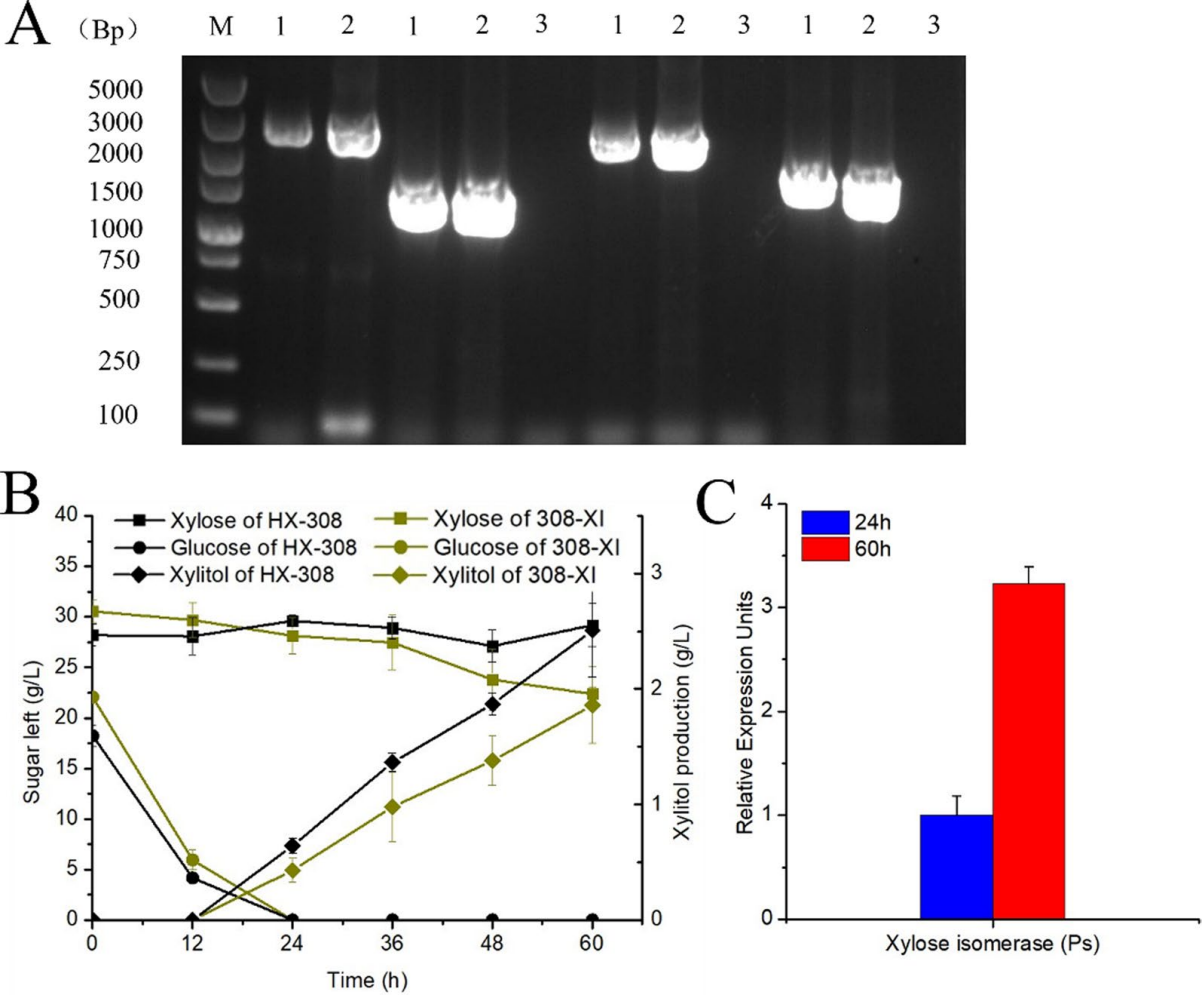
**A** PCR analysis of the 308-XI. 1–2: XI transformant, 3: wild type. **B** Time-course determination of residual glucose (g/L), residual xylose (g/L) and xylitol concentration (g/L) of HX-308 and 308-XI cultivated in medium with 20 g/L glucose and 30 g/L xylose. **C** Quantification of the transcription levels of XI in 24 h and 60 h cultivated in medium with 30 g/L glucose and 20 g/L xylose. 24 h represents the period of glucose consumption and 60 h represents the period of xylose consumption. Data represent the mean ± standard deviation (*n* = 3)

Although a heterologous XDH was successfully introduced, the engineered strain HX-308 was still unable to effectively utilize xylose. To construct a fully functional xylose isomerase pathway, we attempted to introduce a heterologous XI gene. The XI gene from the anaerobic fungus *Piromyces* sp. E2 was reported to have a good expression in *S. cerevisiae*, resulting in stronger enzyme activity than XI genes from most other sources [39, 40]. Therefore, the XI gene of *Piromyces* sp. E2 was introduced into HX-308 to obtain the engineered strain 308-XI (Fig. 3A). When 308-XI was cultured in a medium containing 20 g/l glucose and 30 g/l xylose, the xylose concentration decreased by 8.2 g/l after 60 h (Fig. 3B). And, the 11.3 g/L of DCW was obtained by 308-XI strain, which exceeded that of the wild type (8.81 g/L), indicating that xylose was used to convert into biomass. Furthermore, the mRNA levels of XI were 3.2-fold higher at 60 h (after glucose depletion) than at 24 h (before glucose depletion) (Fig. 3C). This indicates that overexpression of heterologous XI enabled xylose utilization by HX-308.

### Overexpression of heterologous XK, a key limiting enzyme of the xylose metabolic pathway

**Fig. 4:**
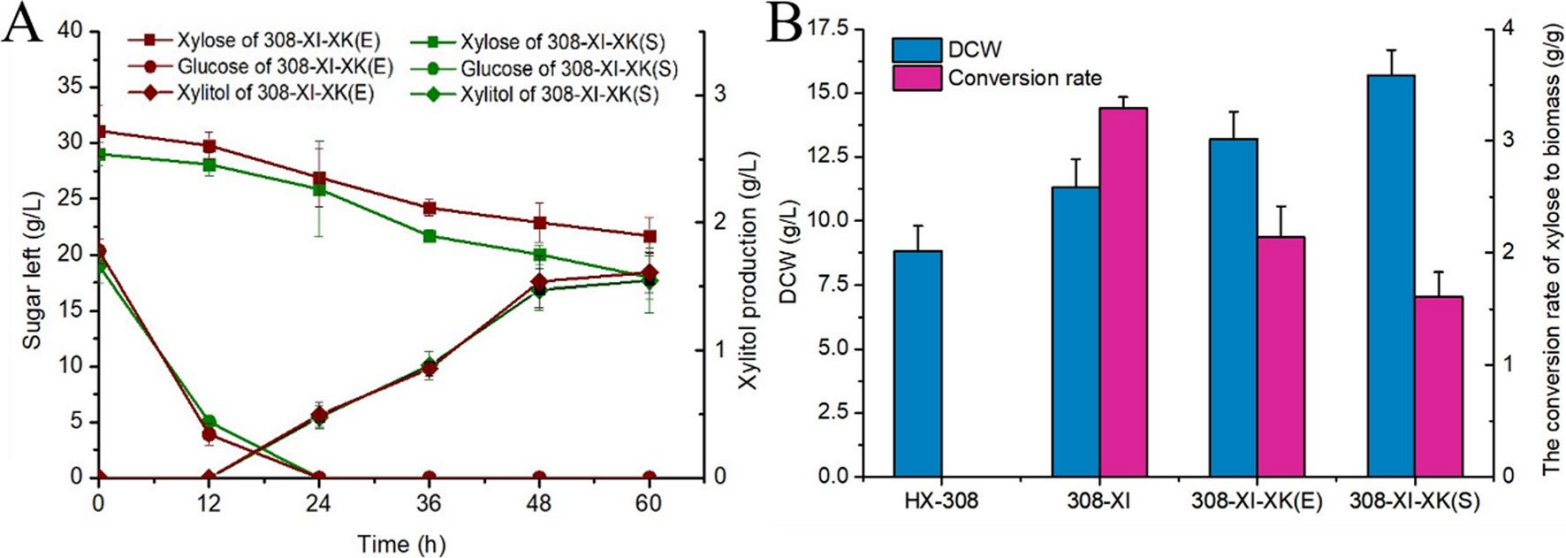
**A** Time-course determination of residual glucose (g/L), residual xylose (g/L) and xylitol concentration (g/L) of 308-XI-XK(S) and 308-XI-XK(E) cultivated in medium with 20 g/L glucose and 30 g/L xylose. **B** The DCW and the conversion rate of xylose to biomass (g xyl /g dcw) for HX-308, 308-XI, 308-XI-XK(S) and 308-XI-XK(E) at 60 h. Data represent the mean ± standard deviation (*n* = 3)

Research has shown that XK may be a critical limiting enzyme in the xylose metabolic pathway. Overexpression of an XK gene in *Y. lipolytica* was a vital step in enabling the engineered strain to grow in a medium containing xylose as the sole carbon source [41]. Similarly, *Thraustochytrid* T18 was unable to grow in a medium with xylose as the sole carbon source until the XK gene from *E. coli* was introduced, combined with the overexpression of its endogenous XI [28]. In order to ensure that XK activity was strong enough to drive the ability of HX-308 to utilize xylose, in addition to native XK gene of HX-308, we selected another two kinds of XK genes from *S. cerevisiae* and *E. coli* that have been reported to possess high activity [28, 42]. Firstly, the catalytic activity of three kinds of XK proteins were tested in vitro, results showed that the activity of two heterologous was stronger than that of native XK (Additional file 1: Fig. S1). The crude enzyme solution containing XK proteins from *S. cerevisiae* and *E. coli* demonstrated an increase in xylose consumption of 24.1% and 28.8%, respectively, in comparison to the crude enzyme solution containing endogenous XK protein. Therefore, the two heterologous XK genes were selected for further genetic engineering. We then overexpressed XI from *Piromyces* sp. and XK from either *S. cerevisiae* or *E. coli* in HX-308, resulting in strains 308-XI-XK(S) and 308-XI-XK(E), respectively. When cultured in the medium containing 20 g/L glucose and 30 g/L xylose, the xylose consumption of 308-XI-XK(S) and 308-XI-XK(E) reached 11.1 and 9.4 g/L at 60 h, respectively (Fig. 4A), which are higher than that of the 308-XI strain, indicating that overexpression of XK further improve xylose utilization. Moreover, as shown in Fig. 4B, the final DCW of three engineered strains was all higher than that of the wild type, and the highest biomass of 308-XI-XK(S) was 15.7 g/L. It is worth mentioning that the conversion rate of xylose to biomass (g xyl/g dcw) by the 308-XI-XK(S) was 1.61, which was lower than that of the 308-XI-XK(E) (2.14 g xyl/g dcw), indicating that the 308-XI-XK(S) could produce more biomass under the condition of consuming the same amount of xylose compared another engineered strain. It has been reported that the conversion rate of xylose to biomass of the engineered *S. cerevisiae* and *Y. lipolytica* have reached a higher level [43–45] due to the abundance of gene editing tool, but it was better than that of *Aurantiochytrium* sp. [26]. Therefore, based on this result, we selected 308-XI-XK(S) for further investigation.

### Carbon source utilization and lipid synthesis in 308-XI-XK(S) grown on corn straw hydrolysate

**Fig. 5:**
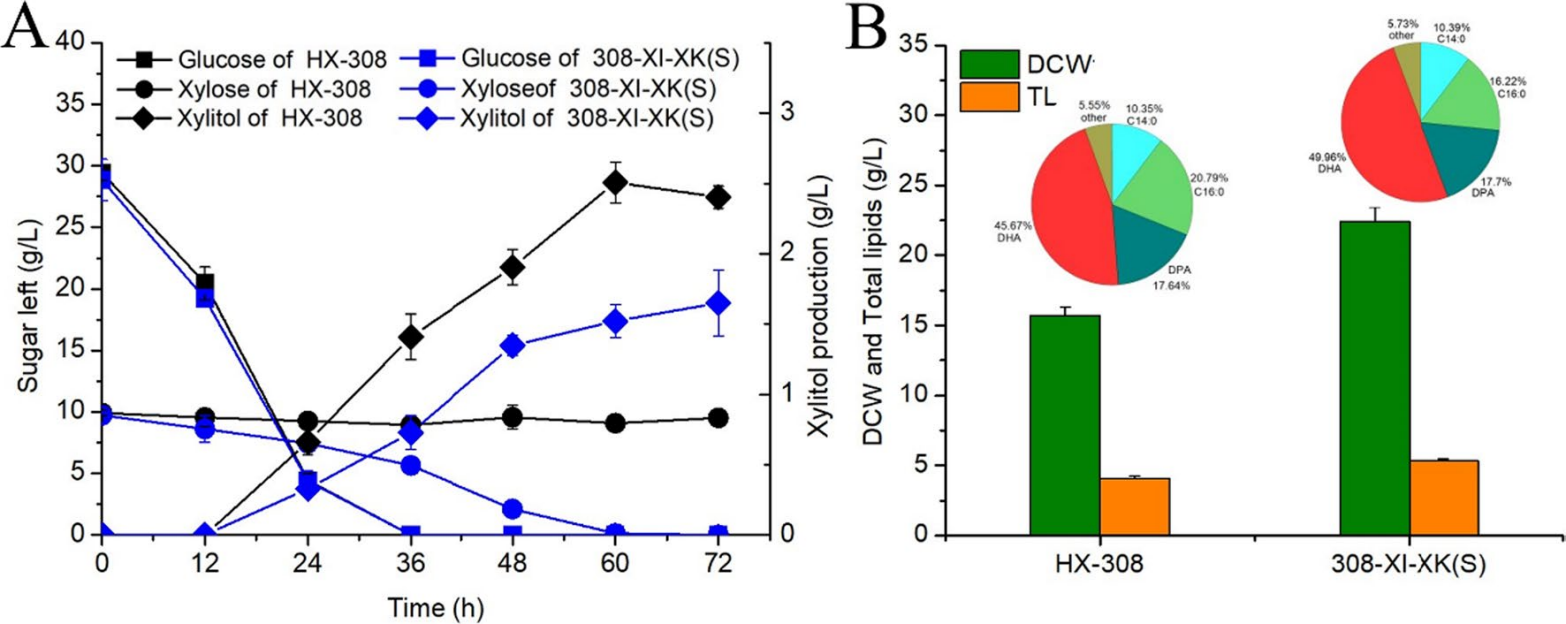
Sugar consumption (**A**), DCW, TL and fatty acid composition (**B**) of HX-308 and 308-XI-XK(S) cultured in corn stover hydrolysate medium for 72 h. *DCW* dry cell weight, *TL* total lipid. Data represent the mean ± standard deviation (*n* = 3)

In order to further investigate the utilization of lignocellulose by 308-XI-XK(S), we conducted fermentation experiments in a medium with corn stover hydrolysate as the main carbon source. In the hydrolysate medium, we first set the initial sugar concentration to 30 g/L glucose and 10 g/L xylose. According to the results, xylose and glucose in the 308-XI-XK(S) medium were totally consumed at 60 h, while there was no significant xylose consumption in the wild type (Fig. 5A). At 60 h, the DCW and total lipids of 308-XI-XK(S) reached 22.4 and 5.3 g/L, representing 42.7 and 30.4% increases over the control strain, respectively (Fig. 5B). This demonstrates that overexpression of XI and XK can enhance the ability of *Schizochytrium* sp. to produce lipids from lignocellulose. A minimal amount of xylitol is still produced in 308-XI-XK(S) medium (Fig. 5A). Therefore, xylitol synthesis gene knockdown can be tried in an attempt to reduce xylose waste. Additionally, the fatty acid profile of 308-XI-XK(S) mainly included 10.4% C14:0, 16.2% C16:0, 17.7% DPA, and 50% DHA, similar to the wild type. Thus, the genetic modification technique used in this study increased lignocellulose utilization without changing the strains' fatty acid profiles.

## Discussion

The cost of the culture medium has a significant impact on the economics of lipid production [46]. In order to lower the cost of fermentation substrates for *Schizochytrium* sp., it is crucial to use cheap carbon sources in place of glucose. To this end, sweet sorghum juice and coconut water have been investigated as fermentation substrates to lower the cost of lipid production [47, 48], but these strategies run the risk of competing with food. By contrast, non-food lignocellulose has been extensively exploited as a low-cost, easily accessible renewable material to lower the cost of fermentation for oleaginous microorganisms. Xylose is one of the main monosaccharides produced after pretreatment of lignocellulose, but most microorganisms cannot utilize xylose, limiting their effective use of lignocellulosic biomass.

The introduction of heterologous genes encoding efficient enzymes of the xylose utilization pathway is a common strategy to engineer microorganisms that cannot utilize xylose. *S. cerevisiae* lacks the enzyme necessary to produce xylulose, a crucial step in the xylose metabolic pathway, and is consequently unable to utilize xylose for growth. However, *S. cerevisiae* was successfully engineered to produce ethanol from xylose by introducing heterologous XR and XDH genes [32]. In fact, whole-genome sequencing and gene annotation of *Schizochytrium* sp. did not find a natural XDH gene. Although the heterologous XDH (PsXDH) was introduced, there was no significant change in xylitol. This phenomenon may be a result of the protein expression being restricted by the transcriptional level [49], that is, PsXDH cannot be efficiently expressed in HX-308. Another possibility is that the PsXDH protein successfully expressed in *Schizochytrium* sp. does not possess the corresponding functional activity. In contrast, the XI pathway makes more efficient use of xylose and reduces the production of xylitol, which is essential. It has been shown that the accumulation of large amounts of xylitol can inhibit normal host growth. When present at higher levels, it can lead to further cell lysis. The XI pathway avoids this phenomenon and reduces the accumulation of xylitol in HX-308 [28]. Additionally, the selection of an XDH with efficient activity in HX-308 is one of the optimal strategies to reduce xylitol and utilize xylose efficiently. Similar strategies have been successfully implemented in the introduction of XI. *S. cerevisiae* was engineered by introducing the XI gene from *Clostridium thermosulfurogenes*, and northern blot analysis revealed that the heterologous XI was efficiently transcribed. However, the recombinant strain was still unable to grow on xylose as the only carbon source [50]. In addition, Parachin et al. [51] enabled *S. cerevisiae* to grow on xylose by introducing a novel XI screened from a soil metagenome library. Therefore, in order to obtain *Schizochytrium* sp. that can utilize xylose, we can attempt to express more XDHs from various sources in *Schizochytrium* sp.

*Schizochytrium* sp. was able to use xylose when heterologous XI was overexpressed, and additional XK overexpression resulted in a further increase of the xylose utilization rate (Fig. 4). It was shown that higher XK activity worked in concert with XI to improve xylose utilization in *Schizochytrium* sp. The isomerase pathway has the advantage of having fewer metabolic steps than the redox pathway, and it is not necessary to consider cofactor balance when engineering strains based on the isomerase pathway. However, none of the strains were able to effectively utilize xylose as the sole carbon source in this study. It has been shown that the gene numbers of XI and XK is critical for the efficient use of xylose for growth. Several studies have shown that the gene numbers of XI and XK is critical for the efficient use of xylose for growth. For example, with increasing amounts of XI and XK, the ability of *Thraustochytrid* T18 to grow in a medium with xylose as the sole carbon source also increased [28]. Similarly, the same phenomenon exists in *S. cerevisiae* [52]. Therefore, this is likely to be the key to limiting the efficient use of xylose by 308-XI-XK(S) for normal cellular metabolism. However, 308-XI-XK(S) still utilizes xylose much less efficiently than glucose, and novel strategies may be needed to obtain *Schizochytrium* sp. strains with improved xylose utilization. Zhou et al. [52] combined genetic engineering and adaptive evolutionary strategies to modify *S. cerevisiae*, yielding a mutant strain with a high xylose utilization rate of 1.866 g/g/h.

Enabling microorganisms to use xylose is essential for the efficient production of bulk chemicals and biofuels from cheap and abundant lignocellulosic biomass. In this study, we improved the lignocellulose utilization of *Schizochytrium* sp. by enhancing its xylose utilization capacity. In addition, *Schizochytrium* sp. strains have recently been engineered for growth on other components present in lignocellulosic materials, such as cellobiose [53]. Future combinations of these approaches might produce more economically viable *Schizochytrium* sp., which would be beneficial not only for large-scale production of microbial lipids, but also for other fermentation products such as squalene, carotenoids, and astaxanthin.

## Conclusions

In this study, we demonstrated that the endogenous XR and XK7938 genes of *Schizochytrium* sp. HX-308 have corresponding functional activities. In addition, after testing xylose metabolism-related genes from different sources, we engineered the strain 308-XI-XK(S), which is capable of efficiently producing lipids from xylose. When 308-XI-XK(S) was cultured in a medium with lignocellulose as the main carbon source, the strain’s xylose utilization rate reached 0.16 g/L/h, while its biomass and lipid production were significantly higher than those of the control. In conclusion, this study provides a reference for further improving the production of lipids by *Schizochytrium* sp. using lignocellulose as a cheap carbon source.

## Materials and methods

### Strains and plasmids

*Schizochytrium* sp. HX-308 (CCTCC M 209059) used in this study was stored in the China Center for Type Culture Collection [54]. *Agrobacterium tumefaciens* AGL-1 and *E. coli* BL21 (DE3) were kind gifts from Dr. Sheng Yang (CAS Center for Excellence in Molecular Plant Science). All plasmids used in this study are listed in Additional file 1: Table S1, and the DNA primers are listed in Additional file 1: Table S2. For efficient expression and execution of enzyme assays, XI, XR, XK3005 and XK7938 proteins were codon optimized for *E. coli* (Additional file 1: Table S4). All plasmids used to express recombinant proteins were constructed based on the pET-24a backbone using Gibson assembly. During plasmid construction, eGFP was fused to the C-terminus of the recombinant protein. The constructed plasmid was introduced into *E. coli* BL21 (DE3) by chemical transformation. All plasmids used to carry DNA elements were constructed based on pZPK-NeoR.

### Culture conditions

Protein expression was conducted as described before [55]. The indicated *E. coli* BL21 (DE3) with recombinant plasmids were first activated in a 3 mL LB liquid medium at 37 °C and 220 rpm for 12 h. Then, 300 μL of the resulting seed culture was used to inoculate 250 mL shake flasks containing 30 mL of TB medium and incubated at 37 °C and 220 rpm. When the cell OD_600_ reached 2–4, IPTG was added to a final concentration of 0.3 mM to induce recombinant protein expression, after which the fermentation was continued at 28 °C. After 24 h, the cells were harvested by centrifugation at 5000 g for 10 min and 4 °C, resuspended in 100 mM Tris–HCl (pH 7.5), and lysed by ultrasonication. The crude cell lysate was centrifuged at 5000 g and 4 °C for 10 min to remove the cell debris and used for enzyme activity assays.

The culture medium and culture conditions for *Schizochytrium* sp. were the same as in our previous study [56]. The main components of *Schizochytrium* sp. medium consisted of glucose 50 g/L, yeast extract 10 g/L, sodium sulfate 12 g/L, magnesium sulfate 2 g/L, ammonium sulfate 4 g/L, potassium chloride 0.7 g/L, calcium chloride 0.2 g/L, sodium glutamate 10 g/L, and potassium dihydrogen phosphate 1 g/L. 1.0 mL of *Schizochytrium* sp. stored in glycerol was inoculated in a shaker containing 50 mL of medium and 250 mL of shaker with a baffle for activation. After 24 h of incubation, the activated cultures were inoculated into 500-mL shake flasks with baffles containing 100 mL of mixed sugar medium for incubation.

### Identification of the xylose metabolism genes

For *Schizochytrium* sp. HX-308, the xylose metabolism genes were obtained by whole-genome sequencing and functional annotation. Further, through BLASTp in NCBI, the translated protein sequences show high identity with xylose metabolic pathway-related enzymes, including XI, XR and XK (NCBI accession number: txid2315210, from *Hondaea fermentalgiana*), whose functions have been identified. The accession numbers of the sequences reported in this article deposited in GenBank: OP428812 (XI), OP428813 (XR), OP428815 (XK3005) OP428814 (XK7938).

### Transformation of HX-308

The transformation method of HX-308 was described previously [57]. Briefly, HX-308 was grown overnight, and then transferred to a fresh medium and incubated until the logarithmic phase. The cells were then collected by centrifuging at 1000 g for 5 min, and washed twice with sterile water. *A. tumefaciens* carrying the target plasmid was grown in YEB medium at 28 °C and 220 rpm for 12 h. The bacterial donor cells were collected by centrifugation at 4000 g for 5 min, resuspended in IM induction medium containing 50 μg/mL kanamycin, and pre-induced at 28 °C and 220 rpm for 8 h. The pre-induced cells were then collected by centrifugation at 4000 g for 5 min and washed twice with sterile water. HX-308 at a concentration of 1 × 10^7^ and *A. tumefaciens* at a concentration of 1 × 10^8^ were mixed and then spread on IM solid medium containing 50 μg/mL kanamycin. After incubation at 28 °C for 48 h, the co-cultures were washed with sterile water, spread on GPYS solid medium containing 300 g/mL cefotaxime sodium and 500 g/mL G418, and incubated for 3 to 5 days to screen for positive transformants. Finally, the presence of the transgene was confirmed by colony PCR.

### Preparation of corn straw hydrolysate

Corn straw hydrolysate was prepared according to Yuan et al. [58]. In a 2 L flask, 500 g of a digestion mixture containing 10% w/w biomass and 2% w/w NaOH was heated at 121 °C for 30 min. After cooling, hydrochloric acid was used to bring the slurry's pH value up to 7.0, and the neutralized biomass was dried in a 60 °C oven until its moisture content was between 10 and 20%. Following pretreatment, cellulase and hemicellulase were used to hydrolyze the biomass at pH 4.8–5.0, 50 °C, and 220 rpm. The hydrolyzed slurry was centrifuged at 6000 g for 10 min after 72 h to obtain a liquid hydrolysate without solids.

### Measurement of glucose, xylose, DCW, total lipid, fatty acids and inhibitor

The supernatant from the centrifuged fermentation broth was used to measure glucose concentrations as described previously [59]. The DCW was determined by gravimetric analysis after centrifuging a volume of fermentation broth at 6000 g for 5 min, removing the supernatant, washing the precipitate twice with water, and then drying in an oven at 60 °C to a constant weight. Lipid analysis was performed as previously reported [60]. Formic acid, acetic acid, and xylose concentrations were determined using a refractory index detector (RID-6A) and an Aminex HPX-87H column maintained at 65 °C. The mobile phase contained 5.0 mM H_2_SO_4_ and flowed at a rate of 0.6 mL/min. Furan and phenolic compounds were analyzed by HPLC using a UV/V is detector (SPD-20A) and a YMC-Pack ODS-A column kept at 35 °C. The mobile phase contained 50% v/v aqueous acetonitrile and had a flow rate of 1.0 mL/min at a detection wavelength of 220 nm. The primary components in the corn straw hydrolysate included the 90.1 g/L glucose, 29.3 g/L xylose, 1.3 g/L vanillin, 1.6 g/L acetic acid, 0.2 g/L furfural, and 0.4 g/L phenol. The conversion rate of xylose to biomass (g xyl /g dcw) for HX-308, 308-XI, 308-XI-XK(S) and 308-XI-XK(E) was calculated according to equation: Xylose consumption/(DCW_20 g/L Glu+30 g/L Xyl_- DCW_20 g/L Glu_).

### Enzyme activity assays with crude cell lysates

The reaction system contained 1 g/L xylose and an appropriate amount of crude enzyme solution. After 12 h at 30 °C, the enzymatic reaction was stopped by boiling for 5 min, and the xylose consumption after was measured by HPLC.

### Quantitative reverse-transcription PCR (qRT-PCR)

To observe changes in transcript levels of heterologous XDH and XI genes, total RNA samples were extracted from cells grown in xylose medium with and without glucose using a fungal RNA mini kit (Omega BioTek, USA). The cDNA synthesis and quantitative real-time RT-PCR (qRT-PCR) analysis were performed by Zoonbio Biotechnology Inc. (Nanjing, China). Gene expression levels were calculated using the 2^−ΔΔCT^ method and normalized to the expression level of glucose-6-phosphate dehydrogenase. The primer sequences used in this study are shown in Additional file 1: Table S2.

## One-sentence summary

The engineering of *Schizochytrium* sp. to efficiently utilize xylose is conducive to improving its utilization of lignocellulose, which can greatly reduce the costs of industrial lipid production.

## Supplementary Information

**Additional file 1: Fig S1**. Xylose content of the crude enzyme solution system before and after incubation. XK7938: xylulose kinase from Schizochytrium sp., XK(E): xylulose kinase from Escherichia coli, XK(S): xylulose kinase from Saccharomyces cerevisiae. Data represent the Kmean±standard deviation (n=3). **Table S1**. Plasmids used in this work. **Table S2**. Primers used in this work. All primers are synthesized by Synbio-tech Co., Ltd., China. **Table S3**. Native xylose metabolic pathway genes of Schizochytrium sp. HX-308. **Table S4**. Xylose metabolism pathway genes from HX-308 after codon optimization in E. coli.

## Data Availability

All data generated or analyzed during this study are included in this published article and its supplementary information files.

## Abbreviations

DCW: Dry cell weight
DPA: Docosapentaenoic acid
DHA: Docosahexaenoic acid
XI: Xylose isomerase
XK: Xylulose kinase
XDH: Xylitol dehydrogenase
XR: Xylose reductase
eGFP: Enhanced green fluorescent protein
