# Supplementary material for: Engineering the xylose metabolism in Schizochytrium sp. to improve the utilization of lignocellulose
Source: Biotechnol Biofuels Bioprod. 2022 Oct 26;15:114. doi: 10.1186/s13068-022-02215-w (PMC9609267; doi:10.1186/s13068-022-02215-w)
Supplement: Supplementary file 1 — Additional file 1: Fig S1. Xylose content of the crude enzyme solution system before and after incubation. XK7938: xylulose kinase from Schizochytrium sp., XK(E): xylulose kinase from Escherichia coli, XK(S): xylulose kinase from Saccharomyces cerevisiae. Data represent the Kmean±standard deviation (n=3). Table S1. Plasmids used in this work. Table S2. Primers used in this work. All primers are synthesized by Synbio-tech Co., Ltd., China. Table S3. Native xylose metabolic pathway genes of Schizochytrium sp. HX-308. Table S4. Xylose metabolism pathway genes from HX-308 after codon optimization in E. coli. [file 13068_2022_2215_MOESM1_ESM.docx]

**Supporting Information for**

**Engineering the xylose metabolism in *Schizochytrium* sp. to improve the utilization of lignocellulose**

Ling-Ru Wang^†a^, Zi-Xu Zhang^†a^, Fang-Tong Nong ^a^, Jin Li ^a^, Peng-Wei Huang ^a^, Wang Ma ^a^, Quan-Yu Zhao^b^, Xiao-Man Sun^a*^

^a^ School of Food Science and Pharmaceutical Engineering, Nanjing Normal University, 2 Xuelin Road, Qixia District, Nanjing, Jiangsu Province, China

^b^ School of Pharmaceutical Science, Nanjing Tech University, No. 30 Puzhu South Road, Pukou District, Nanjing, Jiangsu Province, China

**Correspondence:** [xiaomansun@njnu.edu.cn](mailto:xiaomansun@njnu.edu.cn)

†These authors have contributed equally to this work

Fig S1.


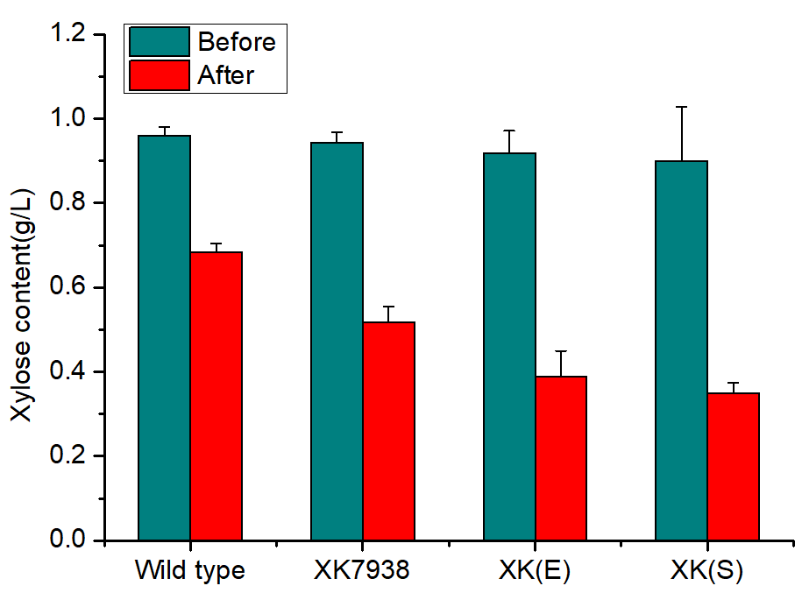


Fig S1. Xylose content of the crude enzyme solution system before and after incubation. XK7938: xylulose kinase from *Schizochytrium* sp., XK(E): xylulose kinase from *Escherichia coli*, XK(S): xylulose kinase from *Saccharomyces cerevisiae*. Data represent the Kmean±standard deviation (n=3).

Table S1 Plasmids used in this work.

| Strains or Plasmids | Characteristics | References |
| --- | --- | --- |
| *Schizochytrium* sp. HX-308 | Wild‑type | Ren et al. Biosystems Engineering 2014,37,865 |
| 308-XDH | HX-308 transformed with pZPK-XDH. Random insertion of XDH. G418‑resistant | This study |
| 308-XI | HX-308 transformed with pZPK-XI. Random insertion of XDH. G418‑resistant | This study |
| 308-XI-XK(E) | HX-308 transformed with pZPK-XI-XK(E). Random insertion of XI-XK(E). G418‑resistant | This study |
| 308-XI-XK(S) | HX-308 transformed with pZPK-XI-XK(S). Random insertion of XI-XK(S). G418‑resistant | This study |
| pET-24a | Kan, T7 lac promoter, his-tag | Novagen |
| PET-24a-XI | The xylose isomerase gene from *Schizochytrium* sp. HX-308 was constructed into pET-24a vector. | This study |
| pET-24a-XR | The xylose reductase gene from *Schizochytrium* sp. HX-308 was constructed into pET-24a vector. | This study |
| pET-24a-XK3005 | The xylulokinase3005 gene from *Schizochytrium* sp. HX-308 was constructed into pET-24a vector. | This study |
| pET-24a-XK7938 | The xylulokinase7938 gene from *Schizochytrium* sp. HX-308 was constructed into pET-24a vector. | This study |
| pET-24a-XI-egfp | The EGFP gene from *Escherichia coli* was constructed into PET-24a-XI vector. | This study |
| pET-24a-XR-egfp | The EGFP gene from *Escherichia coli* was constructed into PET-24a-XRvector. | This study |
| pET-24a-XK7938-egfp | The EGFP gene from *Escherichia coli* was constructed into PET-24a-XK7938 vector. | This study |
| pZPK-NeoR | Kan, P_GAPDH_ promoter, NeoR | Huang et al. Frontiers in Nutrition 2021,8,795651 |
| pZPK-NeoR-XDH | The xylitol dehydrogenase gene from *Pichia Pastoris* was constructed into pZPK-NeoR vector. | This study |
| pZPK-NeoR-XI | The xylose isomerase gene from *Escherichia coli* was constructed into pZPK-NeoR vector. | This study |
| pZPK-NeoR-XI-XK(S) | The xylose kinase gene from *Saccharomyces cerevisiae* was constructed into pZPK-NeoR-XI vector. | This study |
| pZPK-NeoR-XI-XK(E) | The xylose kinase gene from *Escherichia coli* was constructed into pZPK-NeoR-XI vector. | This study |

Table S2 Primers used in this work. All primers are synthesized by Synbio-tech Co., Ltd., China.

| Primers | Sequence (5´-3´) |
| --- | --- |
| PET-R | atgtatatctccttcttaaa |
| PET-F | CTCGAGCACcaccaccaccaccactgagatccggctgctaacaaa |
| XK3005-F | tttaagaaggagatatacatATGAAGGCTAAAAGGGTAGT |
| XK3005-R | gtggtggtggtggtgGTGCTCGAGTTAAGCAACGTTTTGCAGCT |
| XK7938-F | tttaagaaggagatatacatATGGGAATGAGGGGGTCAAA |
| XK7938-R | gtggtggtggtggtgGTGCTCGAGTTACAGTTGGGCTTCGATCT |
| XI-F | tttaagaaggagatatacatATGGCTTCAGGTAATGGAAG |
| XI-R | gtggtggtggtggtgGTGCTCGAGTTAACTGATATAGTGGTTGA |
| XR-F | tttaagaaggagatatacatATGGAAGGGTCAGGATTTGT |
| XR-R | gtggtggtggtggtgGTGCTCGAGTTATTTCAGGCTGGTTGCGC |
| EGFP-R | gtggtggtggtggtgGTGCTCGAGcttgtacagctcgtccatgc |
| EGFP(XI)-F | TCAACCACTATATCAGTTAAatggtgagcaagggcgagga |
| XI(EGFP)-R | tcctcgcccttgctcaccatTTAACTGATATAGTGGTTGA |
| EGFP(XR)-F | GCGCAACCAGCCTGAAATAAatggtgagcaagggcgagga |
| XR(EGFP)-R | tcctcgcccttgctcaccatTTATTTCAGGCTGGTTGCGC |
| EGFP(7938)-F | AGATCGAAGCCCAACTGTAAatggtgagcaagggcgagga |
| 7938(EGFP)-R | tcctcgcccttgctcaccatTTACAGTTGGGCTTCGATCT |
| P _GAPDH_ -F | GATCCAAGCTCAAGCTGCCATTTCTCGACACTTGTCTCCG |
| P _GAPDH_ - R | TGACATAACTAATTACATGAaagcttCTTTTCTCTCGCCTCTCGCT |
| CYC1-F | AGCGAGAGGCGAGAGAAAAGaagcttTCATGTAATTAGTTATGTCA |
| CYC1-R | GAGTCGACCTGCAGCATGCAGCAAATTAAAGCCTTCGAGC |
| XDH(Ps)-F | CAGCGAGAGGCGAGAGAAAAGaatgactgctaacccttccttg |
| XDH(Ps)-R | GTGACATAACTAATTACATGAaagctttactcagggccgtcaatgag |
| XI(P)-F | CAGCGAGAGGCGAGAGAAAAGaatggctaaggaatatttccca |
| XI(P)-R | GTGACATAACTAATTACATGAaagctttattggtacatggcaacaa |
| P-XK-F | CTGTCAAACACTGATAGTTTTTTCTCGACACTTGTCTCCG |
| P-XK(S)-R | ctgaattactgaacacaacattCTTTTCTCTCGCCTCTCGCTG |
| XK(S) -F | CAGCGAGAGGCGAGAGAAAAGaatgttgtgttcagtaattcag |
| XK(S) -R | GTGACATAACTAATTACATGAaagctttagatgagagtcttttccag |
| T-XK(S)-F | ctggaaaagactctcatctaaagcttTCATGTAATTAGTTATGTCAC |
| T-XK-R | TGTCGTTTCCCGCCTTCAGTTTGCAAATTAAAGCCTTCGAGCG |
| P-XK(E)-R | CCCAGCCAGTATTGCGTCATtCTTTTCTCTCGCCTCTCGCT |
| XK(E) -F | AGCGAGAGGCGAGAGAAAAGaATGACGCAATACTGGCTGGG |
| XK(E) -R | TGACATAACTAATTACATGAaagctTCATAATGTGTGCTCCTTAA |
| T-XK(E)-F | TTAAGGAGCACACATTATGAagcttTCATGTAATTAGTTATGTCA |
| XDH-RT-F | atgactgctaacccttcctt |
| XDH-RT-R | ttactcagggccgtcaatga |
| XI-RT-F | atggctaaggaatatttccc |
| XI-RT-R | ttattggtacatggcaacaa |
| G6PDH-RT-F | ATGTTGGACCAAATGATGAG |
| G6PDH-RT-R | TTACTTCTTCTTGGACTCCT |

Table S3 Native xylose metabolic pathway genes of *Schizochytrium* sp. HX-308.

| Gene | sequence (5'-3') |
| --- | --- |
| XI | ATGGCCTCGGGCAATGGAAGCACGAACGGGAGTGGGACCTATTTTCCGAACGTGGATCCCGTCAAGTACGCCGGCCCCGACAGCAGGGACGTGCTAGCGTTTCGATTTTACGACAAGGAGGCGGTGATTTTGGGTAAAAAGATGAAAGACTGGCTCCGGTTTTCTGTCTGTTTCTGGCATACCTTTCGCGGAAGCGGGGCCGACCCCTTTGGGAGACCGACGATCACGCGCCATTTCGAGGGCGACGATGGCACGGACTCCATGGAGAACGCACTTCGTCGCGTGGAGGCAGCCTTTGAGCTTTTCACCAAGCTCGGCGTCGAGTACTACTCGTTTCATGATGTAGACGTGGCCCCAGAAGGCGCGACGATGGCGGAGACTCACGCGAATTTGGAAAAGGTCACGGATCGCATGCTTGAACTGCAAAAGGAGACGGGCGTAAAGCTTCTCTGGGGCACGGCCAATCTTTTTTCGCATCCGCAATATATGAACGGGGCCGCGACCAACCCAGACCCTCTCGTGTTCATGCGCGCTGCGACACAGGTGAAGTTCGCCATGGACGCAACGCACAAACTCGGCGGCGAAGGCTTTGTTTTCTGGGGAGGACGAGAGGGCTACATGCACATTCTCAACACCGACATGGTCCAGGAAATGAACAACTATGCGCGAATGCTCAAGCTTGCCATTGCATACAAGGAGAAGATCGGTTTCTCGGGACAAATCCTTGTGGAGCCCAAGCCTCGCGAGCCCATGAAACATCAGTATGACTATGACGTGCAGACTGTGATTGGTTTTTTACGCGAGCACGGGCTCGAAACCGACGTTCGTCTGAATGTGGAGCCGAACCACACGCAGCTCGCAGGACACGAGTTCGAGCACGATATCGTGCTCGCTGCGAAGCTGGGCATGCTCGGCAGCATTGACGCCAACACCGGGTCAGAGAGCTTGGGTTGGGACACCGATGAGTTCATTACGGACCAGACACGCGCCACAATGCTCTGCAAGGTAATCATTGAAATGGGTGGCTTTCAGCAGGGCGGTCTCAACTTTGACGCCAAAGTTCGACGTGAGAGCACGGACCTTGAAGACTTTTTCATTGCGCACGTTGCGTCAATGGACGCTCTTGCAAAAGGTCTTCGCAATGCAGCCCGCCTCATTTCAGAGGGGCACCTCTCCAGCATGCTTGAGGAACGATATGCAGGCTGGGCTTCCTCTTTAGGGACCCAGATTGGGTCCGGGGAGCTGCCGCTTGATCGCATTGCCGAAATGTACACAACAGTCCACGAGCACGAAGAGAGAAACGGCAAAGCGCACGGAACGACATCAGCGAAGCAAGAAAAGTTCATTGCCGTCTTCAACCACTACATTTCC |
| XR | ATGGAGGGCAGCGGGTTCGTGGAGGTAACCAACGATGCCGCCAAGATGGAGGACGGCAAGGTGTTCCCGCTGACGCTGGGACCGGCGTCCGAGGCGAGCAAGAGCAAACCCGCGGAGACGATCGCGGCGCAGCGCGACCTGGTGCGTGAGCTGGCACGTCAGCACGGAGCAGTGCTGCTTCGCGGCTTTGCGATCGAGAAACCTGAAGACTTTGGAGAGGTCGTCCAGAGCCTCGATCTCGAGAATATGCCGTATGTGGGCGGGGCCGCCGTGCGCCGCAACATTGTTGGCGACTACGTATTTACCGCCAACGAGTCGCCCCCGAGCGAGCCAATCCCGTTTCACCACGAAATGGCTCAGGTGAGCAATCCCCCAGACTATGTCATGTTCTACTGCGAGGTGGCGCCCGAGAGCGGCGGTGAGACGCCGCTCATCCTTTCGCGCAAAGTTTGGTCCTTCTTCGAGCAGAAGTTCCCCGAGGCCGCGCACAAGGTCGAGGAGCTCGGCGTGAAATACGCACGAGTCATGCCCGAGGAAGACGACGAATCGTCAGCGATCGGACGATCGTGGAAAAATACCTTTCTCGTGAGCAACAAGGCCGAGGCTGAGGCCAAGATGCGCGAGATGGGCCACGAGTGGGAATGGCTCCCGAACGGAGACCTCCGTAATGTGACTAACATTGTCCCAGGCGTCCGTGGCGAGCCAGGATCCCCTGGCACCAAAGTGTTCTTCAACTCCATCGTCGCCGCCTTCAAGGGCTGGGTCGATAGCCGCAACGACCCGACCAAGGCCGTCATGTACGGCGATGGTACCTACATTCAATCAGAGGCGCTCGAGGCCGTCGCGGACTTTATGCATCGCGAGCGCGTCTGTGTACCCTGGCAAAAGGGCGACGCAATCATCGTGGACAATCGCGTTGCAATGCACTCGCGCAACACGTACTCGTCACCGCGGCGTGTTTTGGCATCCGTGGGGCGCGTCCCGCTTGCTGGCACCTTTGCCAGCAGCAAAGAGCTCGAGGCCAAGGCCGTTGGCACGCCCGTTCTTGACCGCCTTTACAACGGTGCCTTCATGCCACGGCTCGGTTTCGGACTTTGGAAGCTGCCCAAGGATGTGACTGCCGAGATCGTGCTCAAGGCCATCAAGGCGGGAGTCCGTCACCTTGACTGTGCGTGTGACTATGGCAACGAAAAGGAGGTGGGCGACGGCATTCGCCAGGCCATCAGCGAAGGCATTGTAACCCGTGAGGACCTCTTTGTGGTCTCCAAACTTTGGAACACCTACCATGGTGACAAGGTGCCTGCTGCGCTGACCAAGACGCTCGAAGACCTTCAACTTGACTACGTCGATCTGTACATGATCCACTTTCCGATCCCACAGAAATTTGTCCCTTTCGAAAAGCGCTACCCACCGGAATGGTTCTATGACCCAGACGCCGAAAACCCGCGCGTGGAGCTCGCACGCATTCCCATCGAACGCACGTGGCGCTACATGGAAGCTGAGGTGGTGAGCGGGCGCGCGCGCGCCATTGGTGTTTGCAATTTCTCTGTGCAATTGCTGCGTGACATGATGGCCTATGCCAAGTTCCCTCCCGCCGTTCTCCAGGTCGAGCTTCACCCGCTCAACACCCAAGAGCATCTCGTCGCGTACGCGCGTTCCATGGGTATCAAGGTTATGGCGTTTTCCCCGCTGGGCCACGCAAGTTATGTGGAGATTGGCATGGCTTCGGATGACGATAGTGTTCTGCAAAACGAGCAGGTCAAGGCCATTGCAGACAAGCACGGCAGCACGGTACACCAGGTCGTCTTGCGCTGGGCACTGCAGCGCGGCACATGTGCCGTTTTCAAGTCCTCTAATCCGGATCACATTGTTTCGAACCTCGAGGCCGTGGCTCTGCAGCTAGATGACGAGGAGATGCGCACAATTTCTGCACTCAACAAAAATCGCCGTTTTAATGACCCTGGAGTGTTCTGCGAGGCTGCCTTTAACACCTTTGTGCCCATTTACGACGGTTCTGCCACGAGTCTCAAGTAG |
| XK3005 | ATGAAGGCGAAGCGCGTCGTGGTGGCGGTGGACGTTGGCTCGCGGTCGACCAAGGTCGCAGCCGTGTGCCGGCAAGAGGATGGATCCGAGCAAGTTCTTGCGACGGCGCAAAGTGAGTACGAAGCGCAACATGGGGCTCGCGGCATGGTCAAGCAGGACGCCGAGACGTGGTACACGGCCTTCGTGGACGCGCTGGAGCAGATCCTCCGCAAAGTCGACCCGACGCTATATCGCGTCAGCGAGATTGTCCTGAGTGGTCAGATGCAGTGCGTAGTTTGCGTAGATGGCGGTAGACCGCTCATGCCTGCGATACTGTACTCGGATACGCGAGCGACCGAGGAGGCCGCAGAGCTGACCAAGATCTTTGGCGGCGCCGACAAGATTGCTGCACTCACGGGTAACTTCAAGGACGCAGCTTCTTGCATGGCCAAGGCACTCTGGCTGAGTCGACGCAAGGCTGCAGTATTGACGATTGCGCGTCATGTGCTTTTTGGCGCACATAGTTTCGTCGCTTGGCGACTCACTGGCCGTGCGGCCTGCGATCGTACGACGGCTCAAACAACGAGCTTTAGCAACGAAAATGCCGACGCGTGGTGCGCGGACATTTTTGAGCAAGGCCCGCGCGAACTTGCTGGCGACTGGAAACGACTCATGCCTGATTTGGTAGCCGTCGATGAGCCACTTGCCACGCTCGAAAAAGGACTCATGGGTATTTCAGGGCTTGACTCTACGCTTGCGGCTACCTTTGGTGCGACTCCTGTGACCATTTACCATGGTCCGGGCGATCTCGGAACGACAACGCTGGGCGCCGCGACCGTTCGTGCCCGCGCAGAAGCGGAGGGCGTCCACGTCTCGAAGAGCTACATTTACATTGGCACTTCGGGCTGGATTGCGCGATGCGAGCCAGACAGCGCCAATGCCGCCAGGGAAGCTAATGATGGCGTCTTTCGCATTGCTCACCCACGTTCTGGGATGCAAATTGTTGCCTCCCCGATGACCACGTGTGGAGGCAACGTGGAGTGGATTCGCGCCATGCTCCGGGATCCATCAGGAGGTAAGACAGCATATGCCAAGCTCGACGCACTCGCTGGGTCATGTAACCCTGGCGCAAACGGTGTGTTGTACCTGCCCCATCCGAATGGCGAGCGCGCCCCCTTGCAAAACCCTCATGCTTGTGCTGCTATGGTGGGCATTAGCGGCGAAACAACGCAGGCGCACCTTGCTCGCGCTGTGCTCGAGGGCGTCGCCTTTCATTTACGATGGCTTGCCGAGGCGGGTCACATGTTCGCCGACGCAGACGAAGCTGTCGTCGTTGTGGGCGGCGGAGCGCGCTCGACCGTTTGGTGCCAGATTCTCGCGACGGTGCTAGGACGTCCTGTAGTCGTCAACAAGACAGAAAGCATTGATACAGCTGTGCTTGGCGCTGCGTCCTGCGTCAGCACTGACGAAGAGCCCTCAGCGGCAGCTGCCAAGGGCCTCGTCAAACGTTTCGAGCCGGAAGAGTTTTCTTACAGTGCACTTTATACCGCGTGGCGTGCCGTCTACGACGCTCTTCAAGGCCCTTACGCCGAGCTCCAAAACGTTGCTTGA |
| XK7938 | ATGGGGATGCGTGGGAGCAAAGACGTGTTGTACCTGGGCATCGACGCCGGGACGCAGTCGATCAAGGCAAGCGTGTACGATGGGGCGATGCAGCTCGTCGAGGAGCGCGCGGTCAAGTTTGACGAGGAATTGCCGCAGTACGGTACACGCGCAGGGATGATCGTCGAGGACGAGGCGGCGGGCGTGGTGCGGTCGCCCGTGTTCATGTGGGTCGAGGCGCTCGAAATCGCGCTGGAACGCCTCGCGCAGGCCGTCGACATGTCCAGCATCGTCGCGGTCTCGGGAAGCGCGCAGCAACATTCGAGCGTGTGGTGGCGTGCCTTCCCCGCTAGCTTTGACCCAAACGAGCGCCTTGTGGAGCAGCTGCGCGCTCAAAAGGCCTTTTCCACCGAGCTCTCGCCCATCTGGGCCGACTCGAGCACCTCGGAAGAGGTTAAGAGCGCAGAGGGACGCGTGGGCGGCGCGGCCGCAATGCAGCAGCTCACCGGCTCTGTCGGCACGGAGCGCTTCACGGGAAACCAAATCGCACGCATGGCCAAGAGGCACCCGGAGATCTACAAGGACACGGAGCGCGTAATGTTGCTGAGTACAGCGCTCACGAGCCTCTTTTTGGGCCGTCCTGCCGCCACAGAGGCAGGCGACGCCGCCGGCATGAACATGATGCAGCTCGACAAGAACGTCTGGCTCGACGACCTCGAAGACGTCTTTGACGCCCCGGGTCTTGCGGACAAGGCCGGTGACCTGTGCGAGGCCAACGCCAACTTGGGAGCCGTCTCGGATTTCATGGTGAAGCGCTTTGGCTTTTCGCCCTCGTGCCAGGTTGCTGCTTTCACGGGCGACAATTTGGCCACGCTCGTTGGCTGCGTCGACCGCGCAAATGACGTCATTGTCAGTCTCGGAACCTCGGACACGGTACTTTCCCAGCAGCCTACCCGAAAGCAGGTCAACCAGGCTGCGCTCGTATTTCCGCATCCTACGGATCAAGCGCTCTACATGATTATGATTTGCTACAAGAATGGAGGTGCTGTCCGCAACGACGAAAAGGGCGAAGACCACAAGTGGTCCGAAGTGGACGAGATTCTCCTCGCGGACGCTGATGAGAACGAGCCGGATGTTATTTGCATCTTTTTTCCTCATAAAGAGATTGCGCCACGCCCCATGGACGCCTGCGAGCGAGCCTTTCGCATCGACAATGGCGAAGAGGTCGATGTTGCAAGTCTTTCGTGGCGTCAGCGCGTCCGCGGTGTCATTTTGGCCCGAGCGCTGTCCATCAAGACACACCTCGAGGACTGCAAGTCCTCGTATGATCGTGTTGTCATTACTGGGGGCGGAAGCAACAGCAAGGGAATCCCGGCTGTCTTTCGCCAGGTTCTAGGCGTTCCCGTGTTTACCTCGCAACAGGCCGACGGTGCCTCGCGCGGGGCGGCGCTCAAGGCCATGCTTGCTGTTGACAAAGCGCCCAAGGGTCATGCCATTCTGTCCGAGCGCCCGGAGGCTGCGAGCTTTGCGCAGGTGCCCGCAACGGACCTGTATCGCAAGATCGAGGCGCAGCTG |

Table S4 Xylose metabolism pathway genes from HX-308 after codon optimization in *E. coli*

| Gene | sequence (5'-3') |
| --- | --- |
| XI | ATGGCTTCAGGTAATGGAAGTACAAACGGGTCGGGCACGTACTTCCCGAACGTGGACCCTGTGAAGTACGCCGGTCCGGACAGCCGTGATGTGCTCGCGTTCCGCTTCTACGACAAAGAAGCCGTGATTTTGGGCAAGAAAATGAAAGATTGGCTGCGTTTCTCTGTCTGTTTTTGGCATACCTTTCGTGGTTCTGGCGCGGATCCGTTTGGTCGTCCAACGATCACCCGTCATTTTGAAGGTGATGATGGTACGGACAGCATGGAAAACGCTCTGAGACGTGTTGAGGCTGCGTTTGAGTTATTTACCAAACTGGGTGTTGAATATTACAGCTTCCATGATGTCGATGTAGCACCGGAAGGCGCGACCATGGCAGAAACCCATGCCAACTTGGAGAAGGTTACCGACCGCATGTTGGAGCTGCAAAAAGAGACTGGCGTGAAGTTGCTGTGGGGTACTGCGAACCTGTTTTCCCATCCGCAGTATATGAACGGCGCGGCGACGAATCCGGATCCGCTGGTCTTCATGCGTGCTGCTACGCAAGTGAAATTTGCAATGGATGCGACCCATAAACTCGGTGGTGAAGGTTTCGTGTTCTGGGGTGGTCGTGAGGGCTACATGCACATTCTGAACACCGACATGGTTCAAGAGATGAATAATTATGCGCGTATGCTGAAACTTGCGATCGCGTATAAAGAGAAGATCGGCTTCTCTGGTCAGATTCTTGTTGAACCAAAGCCGCGTGAGCCGATGAAGCACCAGTACGACTATGACGTTCAGACCGTCATTGGTTTCCTGCGCGAGCACGGTCTGGAAACCGACGTTCGTCTGAATGTTGAGCCGAATCACACTCAACTGGCGGGTCACGAGTTCGAGCACGACATCGTTCTGGCTGCTAAGCTAGGTATGCTGGGCAGCATTGATGCTAATACCGGTTCGGAAAGCCTGGGCTGGGACACCGACGAATTTATCACCGACCAGACCCGCGCAACAATGCTTTGCAAAGTGATCATCGAAATGGGTGGCTTCCAGCAGGGTGGCCTGAACTTTGATGCCAAGGTGCGCCGTGAGTCCACCGACCTGGAAGATTTTTTTATCGCACACGTTGCCAGCATGGATGCGTTGGCGAAGGGTTTGCGCAACGCAGCGAGGTTAATTAGCGAAGGCCATCTGAGCAGCATGCTGGAGGAACGTTACGCGGGTTGGGCATCCAGCTTGGGCACCCAAATTGGTTCCGGCGAACTGCCGCTGGATCGTATTGCGGAGATGTACACCACGGTTCATGAGCACGAAGAGCGCAACGGCAAAGCCCACGGCACCACCAGCGCAAAGCAGGAGAAGTTCATCGCGGTGTTCAACCACTATATCAGTTAA |
| XR | ATGGAAGGGTCAGGATTTGTTGAGGTAACAAACGACGCTGCTAAAATGGAAGATGGCAAAGTGTTCCCGTTGACCCTGGGTCCGGCGTCCGAGGCGTCCAAGTCTAAGCCGGCGGAAACCATCGCGGCGCAGCGTGACTTGGTTCGGGAGCTGGCTCGCCAACATGGTGCTGTGTTGCTGAGAGGCTTCGCCATCGAAAAGCCGGAAGACTTCGGCGAAGTGGTTCAGAGCCTCGACTTGGAGAACATGCCGTATGTGGGCGGCGCCGCCGTGCGCCGCAACATCGTTGGCGACTACGTGTTCACCGCGAATGAGAGCCCGCCGAGCGAGCCGATCCCGTTTCATCACGAAATGGCGCAAGTGTCGAACCCGCCGGATTACGTGATGTTTTACTGCGAAGTTGCACCGGAATCTGGTGGTGAAACCCCGCTTATCCTGAGCCGCAAAGTTTGGAGCTTTTTCGAGCAGAAATTCCCGGAAGCGGCACATAAAGTGGAAGAACTGGGTGTTAAGTATGCCCGTGTCATGCCGGAAGAGGACGACGAGTCCAGTGCAATCGGTCGTAGCTGGAAAAATACCTTTCTGGTTTCTAACAAAGCTGAAGCTGAGGCGAAGATGCGTGAAATGGGCCACGAGTGGGAATGGCTGCCGAATGGCGATTTACGTAACGTGACCAATATTGTGCCGGGTGTGCGTGGCGAGCCGGGCAGCCCGGGTACTAAGGTCTTCTTCAACAGCATCGTGGCCGCGTTTAAAGGTTGGGTTGACTCCCGTAACGATCCAACGAAGGCGGTTATGTATGGTGACGGTACGTACATCCAGTCTGAGGCGTTGGAGGCTGTGGCGGACTTCATGCATCGTGAACGTGTGTGTGTTCCGTGGCAGAAGGGTGACGCGATCATCGTGGATAATCGTGTGGCTATGCACAGCCGCAACACGTACAGCTCGCCGCGTCGTGTTCTGGCGTCCGTGGGGCGCGTTCCGCTGGCGGGTACGTTCGCAAGCTCGAAAGAGCTGGAGGCGAAGGCCGTTGGCACTCCGGTTCTGGATCGTCTGTATAACGGTGCCTTCATGCCGCGTCTGGGTTTTGGCCTGTGGAAACTGCCAAAAGATGTGACCGCAGAGATCGTTCTAAAGGCGATTAAAGCGGGTGTACGTCATCTGGATTGTGCGTGCGATTACGGCAACGAGAAGGAGGTTGGTGATGGCATTCGTCAGGCAATTAGCGAAGGTATTGTAACCCGTGAGGACTTGTTTGTTGTAAGCAAGCTGTGGAATACTTACCATGGTGACAAAGTCCCGGCGGCGCTAACCAAAACCCTGGAAGACTTGCAACTGGACTACGTGGATCTGTACATGATTCATTTTCCGATACCGCAGAAATTCGTCCCATTTGAAAAGCGTTACCCGCCTGAGTGGTTTTACGATCCGGATGCTGAGAATCCGCGTGTCGAGCTCGCACGCATTCCCATCGAGCGCACCTGGCGTTATATGGAGGCCGAGGTTGTTTCAGGTCGCGCGCGCGCGATCGGCGTTTGCAATTTTTCCGTGCAGTTGCTGCGTGATATGATGGCATATGCTAAGTTCCCACCGGCGGTGCTTCAGGTTGAACTGCACCCGCTGAACACCCAAGAACACCTGGTTGCCTATGCGAGAAGCATGGGTATCAAGGTGATGGCGTTCAGCCCGTTAGGTCACGCAAGCTATGTGGAAATTGGCATGGCTTCGGATGACGATAGCGTTCTGCAAAACGAGCAAGTTAAGGCGATCGCCGATAAACACGGCAGCACCGTTCACCAGGTGGTTTTGCGCTGGGCACTGCAGCGTGGTACGTGCGCGGTATTTAAGTCCTCCAACCCGGACCACATCGTCTCTAACCTGGAGGCGGTGGCCCTGCAATTGGACGACGAAGAAATGAGAACCATTAGCGCACTCAACAAAAACCGCCGTTTTAATGATCCAGGCGTGTTCTGCGAGGCGGCGTTTAACACCTTCGTGCCGATTTATGACGGCAGCGCAACCAGCCTGAAATAA |
| XK3005 | ATGAAGGCTAAAAGGGTAGTAGTCGCAGTTGATGTTGGTAGCCGCAGCACTAAGGTGGCGGCGGTTTGCCGCCAGGAGGATGGCTCCGAACAAGTGCTCGCGACGGCGCAAAGCGAATATGAAGCGCAGCACGGCGCTCGCGGTATGGTTAAACAGGATGCAGAAACCTGGTATACCGCGTTCGTTGACGCCCTGGAGCAAATTCTGCGTAAAGTCGACCCGACCCTGTACCGCGTGTCCGAGATCGTTCTCAGCGGCCAGATGCAGTGCGTGGTGTGCGTTGACGGTGGCCGTCCGCTGATGCCGGCAATTCTCTACAGCGATACTCGTGCAACCGAAGAAGCAGCGGAGCTGACCAAGATCTTCGGCGGAGCGGACAAAATCGCCGCATTGACCGGTAACTTCAAAGACGCTGCCTCTTGCATGGCGAAGGCGCTGTGGTTGAGCCGTCGCAAGGCAGCGGTCTTGACCATTGCGCGTCATGTTTTGTTTGGTGCGCACTCGTTCGTGGCTTGGCGTCTGACGGGTAGGGCGGCGTGTGACCGTACCACCGCGCAAACCACGAGCTTCAGCAATGAAAACGCAGACGCTTGGTGCGCGGATATCTTCGAGCAGGGCCCGCGTGAACTGGCGGGCGATTGGAAGCGCCTGATGCCGGATCTGGTCGCGGTTGACGAACCACTGGCTACCCTGGAAAAGGGTCTGATGGGGATCTCTGGCCTGGACTCTACCTTGGCGGCGACGTTTGGCGCCACACCGGTCACTATTTACCATGGTCCGGGTGATCTGGGCACCACCACTCTTGGTGCCGCAACCGTACGTGCTCGTGCCGAGGCGGAGGGCGTTCATGTTTCCAAAAGCTATATCTACATCGGTACCAGCGGTTGGATTGCGCGCTGCGAGCCAGACAGCGCTAACGCGGCGCGCGAGGCAAACGACGGCGTGTTTCGTATCGCACACCCTCGTTCTGGCATGCAAATCGTGGCCTCACCGATGACCACCTGTGGTGGCAACGTGGAATGGATTCGTGCCATGCTGCGCGATCCGTCCGGTGGTAAAACGGCGTATGCGAAGCTGGACGCTCTGGCGGGTAGCTGTAATCCAGGCGCGAACGGTGTCTTATACCTGCCGCATCCGAATGGTGAACGTGCTCCGTTGCAAAACCCGCACGCCTGCGCAGCCATGGTGGGCATTAGCGGTGAGACGACCCAGGCACACCTGGCTAGAGCGGTGTTGGAGGGTGTTGCGTTCCACCTGCGTTGGCTGGCAGAAGCGGGTCATATGTTTGCTGACGCGGACGAGGCAGTTGTGGTAGTCGGAGGCGGCGCGCGTAGCACCGTGTGGTGTCAGATCCTCGCGACCGTTTTGGGCCGTCCGGTGGTGGTTAATAAAACGGAATCGATTGATACGGCCGTTCTGGGTGCCGCGAGCTGCGTGTCCACCGATGAGGAGCCGTCAGCTGCTGCTGCGAAGGGTCTGGTGAAACGTTTTGAGCCGGAAGAATTTTCCTACAGCGCGCTGTACACCGCTTGGCGTGCGGTGTATGATGCGTTACAGGGTCCGTATGCAGAGCTGCAAAACGTTGCTTAA |
| XK7938 | ATGGGAATGAGGGGGTCAAAAGATGTACTATACCTCGGCATTGATGCAGGCACGCAAAGCATTAAAGCGAGCGTTTACGACGGCGCCATGCAGTTGGTGGAGGAGAGGGCGGTCAAGTTCGATGAAGAATTACCGCAGTATGGAACGCGTGCCGGCATGATCGTGGAAGACGAGGCAGCTGGTGTGGTTCGTAGCCCGGTTTTCATGTGGGTTGAGGCGCTGGAGATCGCGTTGGAGCGCCTTGCTCAAGCGGTGGATATGAGCTCTATCGTTGCTGTCTCTGGTAGCGCACAGCAGCATAGCTCTGTGTGGTGGCGTGCGTTTCCGGCGTCGTTCGACCCGAATGAACGCCTGGTCGAGCAGCTGCGTGCGCAGAAAGCCTTCTCCACCGAGCTGTCCCCGATTTGGGCAGACAGCAGCACCTCCGAGGAAGTCAAGTCTGCCGAAGGTCGCGTGGGTGGCGCTGCTGCTATGCAACAACTAACCGGCTCCGTTGGTACTGAGCGCTTTACCGGTAATCAAATTGCACGCATGGCAAAACGTCATCCGGAAATTTATAAAGATACCGAACGTGTAATGCTGTTGTCTACGGCTTTGACCTCCCTGTTTCTGGGTCGTCCGGCGGCGACCGAAGCGGGCGATGCTGCTGGCATGAATATGATGCAGTTGGACAAGAACGTTTGGCTGGATGATCTGGAGGACGTATTTGACGCGCCTGGTCTGGCCGACAAGGCGGGCGATCTTTGCGAGGCGAACGCGAACCTGGGCGCTGTGTCGGACTTCATGGTTAAGCGCTTTGGTTTTTCGCCGAGCTGTCAGGTTGCGGCGTTCACCGGCGACAACCTGGCTACCCTGGTTGGCTGCGTGGACCGTGCAAACGATGTGATCGTGAGCTTGGGCACAAGCGATACCGTTCTGAGCCAGCAGCCGACTCGCAAGCAGGTTAACCAGGCGGCTTTGGTGTTCCCGCACCCGACGGATCAGGCCCTGTACATGATCATGATTTGCTACAAGAACGGAGGTGCGGTTCGCAATGATGAGAAAGGTGAAGACCACAAATGGAGCGAAGTTGACGAGATCCTTCTGGCGGACGCGGATGAGAACGAACCGGATGTGATCTGCATTTTTTTCCCGCACAAAGAAATCGCGCCACGTCCGATGGATGCCTGCGAACGTGCATTCCGTATTGATAACGGTGAAGAGGTTGACGTGGCGAGCCTGAGCTGGCGTCAACGTGTTAGAGGCGTTATTCTGGCGCGTGCCTTGTCCATCAAGACCCACCTGGAGGACTGTAAAAGCTCCTATGATCGTGTCGTGATCACCGGTGGTGGTAGCAATAGCAAAGGTATCCCGGCGGTCTTCCGCCAGGTACTCGGCGTGCCGGTCTTCACCTCGCAACAAGCAGATGGTGCCAGCCGTGGTGCGGCGCTGAAAGCCATGTTAGCGGTGGACAAGGCTCCAAAAGGTCATGCAATTCTATCAGAACGCCCGGAGGCGGCGAGCTTTGCACAAGTGCCGGCGACCGACCTGTACCGTAAGATCGAAGCCCAACTGTAA |
